# Electrosprayed Chitosan–Calcium Phosphate Microshell Composite Coatings on Silanized Titanium Substrates

**DOI:** 10.3390/jfb17080409

**Published:** 2026-08-17

**Authors:** Andrew Blass Watson, Matthew J. Atwill, Tomoko Fujiwara, Ranganathan Gopalakrishnan, Jessica Amber Jennings, Joel D. Bumgardner

**Affiliations:** 1 University of Memphis-University of Tennessee Health Science Center Joint Graduate Program in BioMedical Engineering, Biomedical Engineering Department, The University of Memphis, Memphis, TN 38152, USA or atwillm2017@gmail.com (M.J.A.); jjnnings@memphis.edu (J.A.J.); 2 Department of Chemistry, The University of Memphis, Memphis, TN 38152, USA; tfjiwara@memphis.edu; 3 Mechanical Engineering Department, The University of Memphis, Memphis, TN 38152, USA; rgplkrsh@memphis.edu

**Keywords:** chitosan, chitosan–calcium phosphate composite, implant coatings, electrospray, silanization

## Abstract

Chitosan and calcium phosphate (CaP) are attractive bioactive coating materials for titanium (Ti) orthopedic implants, but coating approaches must provide uniform deposition, adequate adhesion, and cytocompatibility. This proof-of-concept study evaluated whether CaP microshells could be incorporated into electrosprayed chitosan coatings bonded to silanized Ti substrates without compromising coating properties. CaP microshells were synthesized using carbon microsphere templates and added to chitosan electrospray solutions at 0.25, 0.5, and 1.0 wt% relative to chitosan. Electrospray parameters were adjusted, and coatings were characterized by scanning electron microscopy, energy dispersive X-ray spectroscopy, Fourier transform infrared spectroscopy, tensile adhesion testing, water contact angle measurements, and W-20-17 bone marrow stromal cell culture. Increasing capillary diameter and reducing pressure enabled stable deposition of uniform composite coatings containing up to 1.0 wt% CaP. CaP microshells were distributed across the coating surfaces and throughout the coating thickness. Silanization significantly increased coating adhesion compared with non-silanized Ti, while CaP incorporation up to 1.0 wt% did not significantly reduce bond strength. All coatings were hydrophilic and supported viable cell attachment and growth over five days. These findings support the feasibility of electrosprayed chitosan–CaP microshell coatings as adhesive, cytocompatible bioactive coating platforms for Ti implant materials.

## 1. Introduction

Arthritis is the leading cause of disability in the U.S., and, according to the Center of Disease Control (CDC), between 2013–2015, an estimated 54.4 million US adults (22.7%) and 300,000 juveniles were diagnosed with arthritis [1,2,3]. In patients with severe arthritis, ongoing pain, limited function, and diminished life quality, biomedical total joint implant devices have become a reliable and effective means for replacing and restoring joint function. Approximately 2 million total knee and total hip arthroplasties are estimated to be performed annually [4,5,6].

While most of these orthopedic implants perform well during their operational lifetimes, reports indicate that about 5% of implants experience some type of failure [1,7,8,9,10]. Common causes of failure include mechanical loosening, infection, and dislocation [8,9,10]. Early fixation of implants in bone has been identified as a critical step in obtaining long-term success of total hip replacement [4,7,8,9,10,11,12]. Ti is widely used for fabricating components of orthopedic implant devices due to its capacity for osseointegration, as well as its excellent corrosion resistance and high fatigue limit [13]. To further improve osseointegration of Ti implants, surface coatings using different materials, including calcium phosphates, bioglass, and polymers such as chitosan, have been explored [14,15,16,17,18,19,20,21,22,23,24,25,26,27,28,29].

Calcium phosphate (CaP) is a highly biocompatible inorganic biomaterial. CaP coatings have been extensively investigated as implant coatings due to their similarity to native calcium phosphate mineral found in bone [14,15,16]. CaP coatings have demonstrated excellent biocompatibility, osteoconduction, and osseointegration but are observed to be brittle, suffer delamination problems, and can exhibit higher rates of bacterial infection compared to non-CaP-coated implants, limiting their use clinically [14,15,16]. In addition, calcium phosphate coatings made by traditional sputter coating, plasma spraying, or other high-energy coating methods have limited ability to incorporate bioactive agents including antimicrobial and growth factors [14,15,16].

Bioglass has also been used as a coating. Bioglass is a silicate-based (SiO2-CaO-P2O5) bioactive glass that, when implanted, forms a hydroxyapatite layer on the bioglass surface [17,18,19]. Studies have reported that coatings of bioglass on metallic and ceramic implants improve the rate of osseointegration [17,18,19]. However, bioglass coatings can be brittle and susceptible to instability or delamination when exposed to large mechanical loads; therefore, their clinical use has been limited to non-load-bearing applications [19]. This limitation highlights the need for coating systems to maintain interfacial stability and coating adhesion to meet implant mechanical loading needs [19].

Natural polymers have been used in medical applications for decades thanks to their biocompatibility, biodegradability, and tunable properties. Chitosan, a linear polysaccharide derived from the deacetylation of chitin polymer, has attracted interest as an implant coating material due to its biocompatibility, biodegradability, bacteriostatic properties, and ability to serve as a bioactive coating platform [30,31,32]. Many techniques have been used to fabricate chitosan coatings, including solution casting with silane bonding, sputter coating, freeze-drying, layer-by-layer electrolytic deposition, and electrospraying [20,21,22,23,24,25,26,27,28,29]. These coating methods have been reported to create chitosan coatings that are able to support bone cell attachment, growth and differentiation, osseointegrate in animal models and/or incorporate bioactive agents in vitro and in vivo. However, each method has its drawbacks. Solution casting makes it difficult to coat complex shapes uniformly. High-energy coating methods, such as sputter coating, can damage or limit incorporation of bioactive agents. Layer-by-layer electrolytic deposition requires large volumes of coating solution, which can be wasteful and add expense.

Electrospraying is a method, based on the principle of particle/droplet charging, that overcomes many of the limitations of other chitosan coating fabrication methods. The advantages of electrospraying include the ability to coat 3D surfaces with complex and irregular contour by utilizing electric charge to give precise control over coating thickness and reduced waste of coating material compared with solution casting and other electrophoretic deposition methods [26,33,34,35]. Furthermore, electrospraying can incorporate bioactive molecules or other coating components under comparatively mild processing conditions [33,34,35,36,37]. Several studies have investigated electrospray deposition methods to create chitosan-based coatings on implant materials to improve osseointegration and enable localized incorporation of bioactive components [26,33,34,35,36,37].

The aim of this proof-of-concept study was to fabricate an electrosprayed chitosan–CaP microshell composite coating on silanized Ti substrates and evaluate whether CaP microshells could be incorporated without compromising coating uniformity, adhesion, wettability, or preliminary cytocompatibility. The silanization method was selected since silane molecules can act as linkers to bond chitosan to titanium surfaces to improve coating attachment and stability. The novelty of this work is the combined use of silanized Ti substrates, electrosprayed chitosan, and CaP microshell structures within one composite coating architecture. Composite coatings were compared to plain chitosan-coated and uncoated Ti controls for changes in hydrophobic/hydrophilic properties, adhesive strength, and ability to support bone cell attachment and growth, and they were also evaluated in terms of distribution of CaP particles within the electrosprayed coatings.

## 2. Materials and Methods

Titanium (Titanium Industries, commercially pure ASTM F67 grade 2, Hillsboro, TX, USA) was used as the substrate for fabricating electrosprayed coatings. Tri-ethoxy-silylbutyraldhyde (TESBA) silane, purchased from Gelest (product code: SIT8185.3, Morrisville, PA, USA), was used for bonding the electrosprayed coating to the titanium surface. Carbon microspheres were obtained from the Physics Department of the University of Memphis. Chitosan powder (92.6 DDA, molecular weight 300–700 kDA, product no. 24711), purchased from Heppe Medical Chitosan GmbH, Halle, Germany, was used for making the chitosan coatings. All other reagents and chemicals used were obtained from Thermo Fisher Scientific, Waltham, MA, USA.

### 2.1. Sample Preparation

#### 2.1.1. Cleaning and Hydroxylation of Titanium Substrate

To create electrosprayed coatings bonded to the titanium, bifunctional TESBA silane molecules were used as previously described [22]. Briefly, in the reaction scheme, the triethoxysilyl end of the TESBA molecule is reacted via condensation with hydroxyl groups on the titanium surface to create covalent -Si-O-Ti (siloxane) linkages. Then, the butyraldehyde end of the TESBA molecule is reacted with amine (-NH2) groups on the chitosan polymer to form covalent imine bonds (-C=N-). Thus, the TESBA molecule serves as a linker forming a covalent bond between the titanium surface and the chitosan coating.

To prepare the hydroxylated surfaces for reacting with TESBA silane, Ti substrates were wet-ground with a sequence of 400, 600, 800, and 1200 grit silicon carbide grinding paper and then cleaned with dilute soap and warm water. Next, the substrates were ultrasonically (FS60H, Thermo Fisher Scientific, Waltham, MA, USA) cleaned with acetone, 100% ethanol, and deionized water for 10 min each. After cleaning, samples were submerged in 5 M sodium hydroxide (NaOH) at 60 °C for 24 h to enhance surface hydroxide formation [22]. Samples were washed in fresh deionized distilled water (DI) water and stored in DI water to prevent drying until the silanization step [22].

#### 2.1.2. Silanization of Titanium Substrate

The silanization process of the Ti substrate was adopted from prior studies [20,21,22,23,24,25,26]. First, Ti substrates were placed in a glass Pyrex dish on a magnetic stir plate and stirred in a 5:95 (*v*/*v*) deionized water/ethanol solution (pH = 4.5). The solution pH was maintained at 4.5 using a pH meter (Accumet BASIC, AB15 pH meter, Thermo Fisher Scientific) and 1M acetic acid and 1M sodium hydroxide solution [26]. Tri-ethoxy-silylbutyraldhyde (TESBA) silane was added to make a 2% (*v*/*v*) silane solution in ethanol. The Ti substrates were placed on a belly dancer (IBI Scientific, The Belly Dancer, Thermo Fisher Scientific, Waltham, MA, USA) for 10 min to allow hydrolyzed ethoxy groups of the silane molecules to interact with hydroxyl groups on the Ti substrate surface through hydrogen bonding. The silanized substrates were then rinsed with ethanol to remove non-adhered silane and cured at 110 °C in an oven (Thermo Fisher Scientific, Waltham, MA, USA) for 10 min to convert hydrogen bonds between silane and Ti to covalent Si-O-Ti bonds. The silanized substrates were immediately stored in a vacuum chamber after curing.

#### 2.1.3. Chitosan Solution Preparation

For electrospraying, 3 parts of a 1 wt% chitosan in 0.5 v% acetic acid solution were mixed with 1 part of a 95 v% ethanol/5 v% 2-propanol solution. The alcohol solution was used to reduce the viscosity and surface tension of the chitosan solution to make it sprayable [26]. The solution was stirred using a magnetic stirrer to ensure thorough mixing. After mixing, the pH of the solution was adjusted to 5.1 and then sonicated for 7 min (Sonic probe, CL-18, Thermo Fisher Scientific, Waltham, MA, USA) to remove microbubbles in the solution before spraying [26].

#### 2.1.4. Synthesis of CaP Microshell Particles

The procedure for the synthesis of the CaP microshells was adapted from Wu et al. [38]. Briefly, carbon microspheres (diameter = 0.8 µm) served as sacrificial precursor templates and nucleation sites for CaP deposition. The carbon microspheres were incubated (1.5 mg/mL) at 37 °C in simulated body fluid with the pH adjusted to 7.4 using tris(hydroxymethyl)aminomethane base and hydrochloric acid, as described by Kokubo et al. [39]. After approximately 4 days, the precipitated particles were collected and rinsed in distilled water until they turned grey. The particles were then dried, ground (Cole-Parmer Mortar and Pestle), and baked in oven at 500 °C for 5 h to remove the carbon microsphere precursors from the final CaP shells [38]. The CaP shell particles obtained were stored in an airtight container until further use.

#### 2.1.5. Incorporation of CaP Shells into Chitosan Solution

Calcium phosphate shells were incorporated into the chitosan electrospraying solution at 0.25, 0.5, and 1.0 wt% of CaP shells to wt% chitosan powder. Solutions were ultrasonicated (Sonic probe, CL-18, Thermo Fisher Scientific, Waltham, MA, USA) for 5 min using an ultrasonic probe to break apart any CaP aggregates in the solution and then vortexed to ensure homogenous mixing of CaP shells in the solution prior to spraying.

#### 2.1.6. Electrospraying Parameters

The initial spray parameters used were adapted from previously developed chitosan electrospraying conditions using the same in-house custom electrospraying setup [26]. These parameters were subsequently modified to achieve spray flowrate, spray stability, and spray conditions of the chitosan–CaP microshell suspension and to produce a stable, uniform coating on Ti surfaces. Spray flow rate refers to the rate of volume of chitosan solution ejected from the electrospray apparatus per unit time, and spray conditions refer to dry or wet deposition of chitosan droplets on the substrate. Spray stability refers to the ability of the electrospray to maintain uninterrupted spraying without large droplet formation or solution accumulation on the nozzle. Parameter tuning followed a defined, literature-guided iterative workflow in which capillary size, pressure, voltage, and substratecapillary distance were adjusted sequentially after CaP microshells were added to the spray solution. After spraying, coatings were neutralized in a 0.25 M phosphate buffer solution, rinsed with DI water and dried at room temperature in a laminar flow hood.

### 2.2. Characterization

#### 2.2.1. Particle Size and Charge Determination

The particle size distribution of CaP microshells was measured using dynamic light scattering (Nano-ZS, Malvern Instruments, Malvern, UK). From the measured distributions, Z-average diameters of the shells were calculated. A micro-electrophoresis device (Nano-ZS, Malvern Instruments, Malvern, UK) was used to determine the surface charge of the CaP shells and acquire the zeta potential. For both the measurements, the samples were diluted with deionized water to avoid or reduce the multiple scattering effects.

#### 2.2.2. Shape, Surface Morphology and Phase of CaP Shells

To determine surface morphology and elemental composition, prepared CaP shells were examined by SEM and EDS using scanning electron microscopy (SEM) (NovaNanoSEM650, FEI/ThermoFisher Scientific, Waltham, MA, USA), [26]. Briefly, for SEM/EDS, the shells were evenly mounted on to the metallic stub and sputter coated with gold–palladium. SEM images and EDS spectra were recorded at an acceleration voltage of 15–18 kV. X-ray diffraction was used to determine identify the composition and presence of crystalline phases in the CaP shells. Spectra were recorded in the angular range of 2θ = 15–50°. For these experiments, an Empyrean (Malvern Panalytical, Malvern, UK) diffractometer (45 kV and 40 mA) with copper Kα1 radiation (λ = 1.540598 Å) was used.

#### 2.2.3. Determination of Silane Deposition on Titanium Samples

Fourier transform infrared spectroscopy (FTIR, Perkin Elmer Frontier, Waltham, MA, USA) in attenuated total reflectance (ATR) mode was used to examine the surface chemistry and bonding of silane and chitosan to the Ti surface. Samples were examined with clean polished Ti as a baseline after sodium hydroxide (NaOH) treatment to verify hydroxylation and then after silanization treatment to verify silane deposition.

#### 2.2.4. Electrospraying Parameter Determination

The initial starting parameters for electrospraying chitosan are shown in Table 1. The parameters were adjusted sequentially to determine which variables were critical for stable coating formation. The adjustment order was capillary size, pressure, voltage, and substrate–capillary distance.

#### 2.2.5. Determination of Surface Morphology of Chitosan Coatings Loaded with CaP Shells

To confirm and evaluate the incorporation of CaP shells into the chitosan coatings, the samples were examined using SEM and EDS. SEM was used to examine surface morphology of the coatings with and without CaP shell particles. Coatings were further evaluated in SEM using EDS to map the Ca and P distribution as an indicator of CaP shell particle distribution in coatings. Samples were also evaluated from the edge using SEM and EDS to examine the CaP distribution throughout the coating thickness. Edge views were obtained by cutting a cross-section of a coated sample and placing it on its side in the SEM.

#### 2.2.6. Determination of Bond Strength of the Chitosan Coating

To test the bond strength of the coatings to the Ti surfaces, aluminum studs were attached to the coatings using GorillaWeld Steal Bond epoxy (Gorilla Glue, Cincinnati, OH, USA) [26]. Glued specimens were loaded into an Instron 33R test frame (Series 4465, Instron, Norwood, MA, USA) using custom fixtures and tested in terms of tension using a 5 kN load cell at 0.5 mm/min until failure. The maximum tensile stress before failure was recorded. The test specimens were examined visually to determine the failure mode as either adhesive failure of the glue, adhesive failure at the coating–substrate interface, or cohesive failure of the coating. The test groups were control groups of electrosprayed coatings on silanized and non-silanized Ti and electrosprayed coatings with 0.25, 0.5, and 1.0 wt% CaP on silanized Ti. The 95% confidence intervals were calculated, and ANOVA with post hoc testing using the Holm–Sidak method was used for statistical comparison. All groups were compared to each other.

#### 2.2.7. Determination of Hydrophilic Nature of Chitosan Coating

To assess the change in hydrophilic or hydrophobic properties of the chitosan coating after incorporation of CaP shells, water contact angle measurements were performed using contact angle measurement (CAM) (VCA Optima, AST Products, INC, Billerica, MA, USA). Contact angle measurements were performed on chitosan coatings of 0.0, 0.25, 0.5, and 1.0 wt% CaP electrosprayed onto silanized Ti specimens. The water droplet size was 3 μL for all the samples. Four replicate (*n* = 4) samples per test group were evaluated. The 95% confidence intervals of the angles were calculated for statistical comparison.

#### 2.2.8. In Vitro Cytocompatibility Studies of the Chitosan Coatings

A cytocompatibility study was performed on the chitosan coatings using W-20-17, ((W-20 clone 17] (ATCC^®^ CRL-2623™, Manassas, VA, USA)) mouse bone marrow stromal cells (BMSCs) with osteogenic differentiation potential. The cells were subcultured in high-glucose Dulbecco’s modified Eagle’s medium (DMEM) with 10% heat-inactivated fetal bovine serum (FBS), 50 µg/mL penicillin, 50 µg/mL streptomycin, and 100 µg/mL neomycin at 37 °C in a 5% CO_2_ incubator. Cells were seeded on control (0 wt% CaP) and test chitosan–CaP-coated specimens (9.5 mm diameter disks) in 48 well plates at 3 × 10^4^ cells/well in 0.5 mL of medium and allowed to attach overnight. Medium was removed and replaced with fresh medium every 2–3 days. Viability was measured 1, 3, and 5 days after seeding using a CellTiter-Glo assay (CellTiter-Glo^®^ Luminescent Cell Viability Assay, Cat. # G7570, Promega, Fitchburg, WI, USA). The Cell Titer Glo assay is a homogeneous method of determining the number of viable cells in culture based on luminescent quantitation of the ATP present. Data were recorded as relative luminescence units. There were three replicates per group for each time point. Data were then analyzed statistically using two-factor ANOVA followed by Tukey’s post hoc testing (α = 0.05 level of significance).

## 3. Results

### 3.1. Size, Charge Determination, Crystallinity and Surface Morphology of CaP Mircoshells

**Figure 1:**
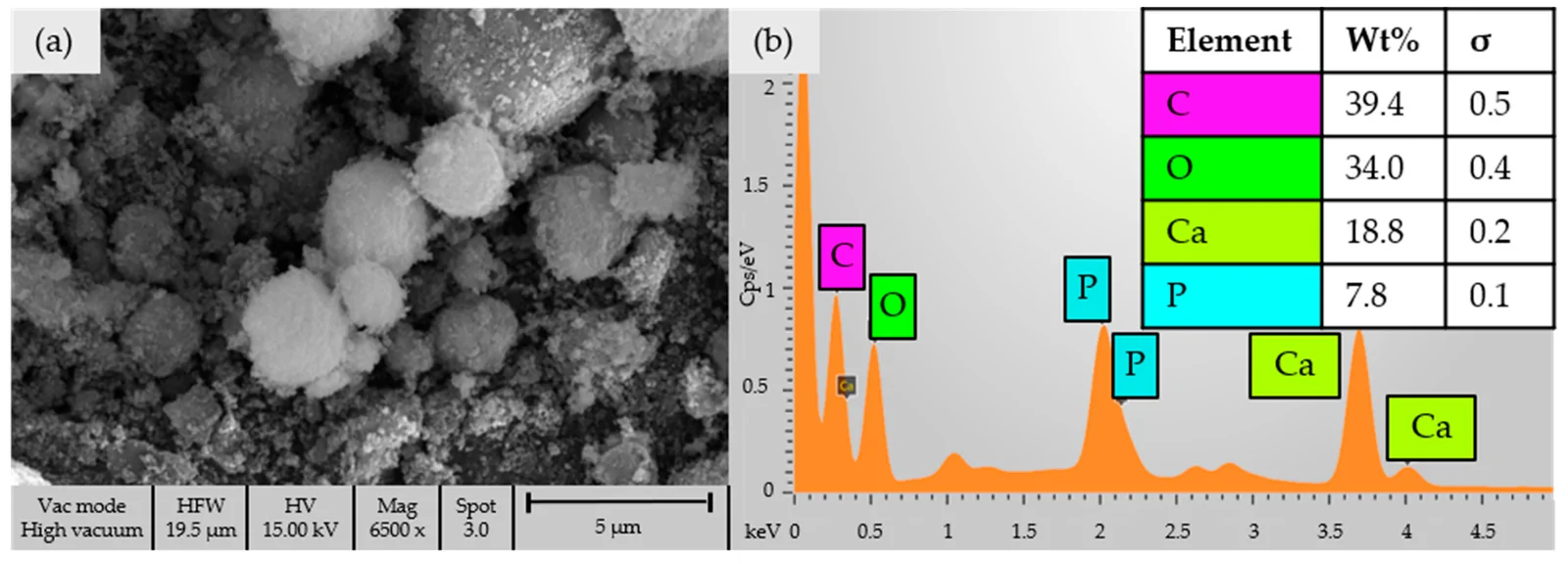
(**a**) SEM image of calcium phosphate shell particles; (**b**) EDS spectra showing major peaks for Ca, P and O associated with calcium phosphate composition. Small non-labeled peaks for Na (~1.04 and 1.07 keV) and Cl (~2.62 and 2.82 keV) were also observed.

The CaP particles produced in this work were spherical microshells with rough surfaces composed of lathlike apatite crystallites (Figure 1a). EDS spectra confirmed the presences of Ca and P, although small amounts of Na and Cl from the aqueous process used to manufacture particles were also detected (Figure 1b). The particles had diameters ranging from 1.1 to 2 µm, a surface charge of +26.28 mV, and an electrophoretic mobility of 2.34 ×10^−4^ cm^2^/Vs.

**Figure 2:**
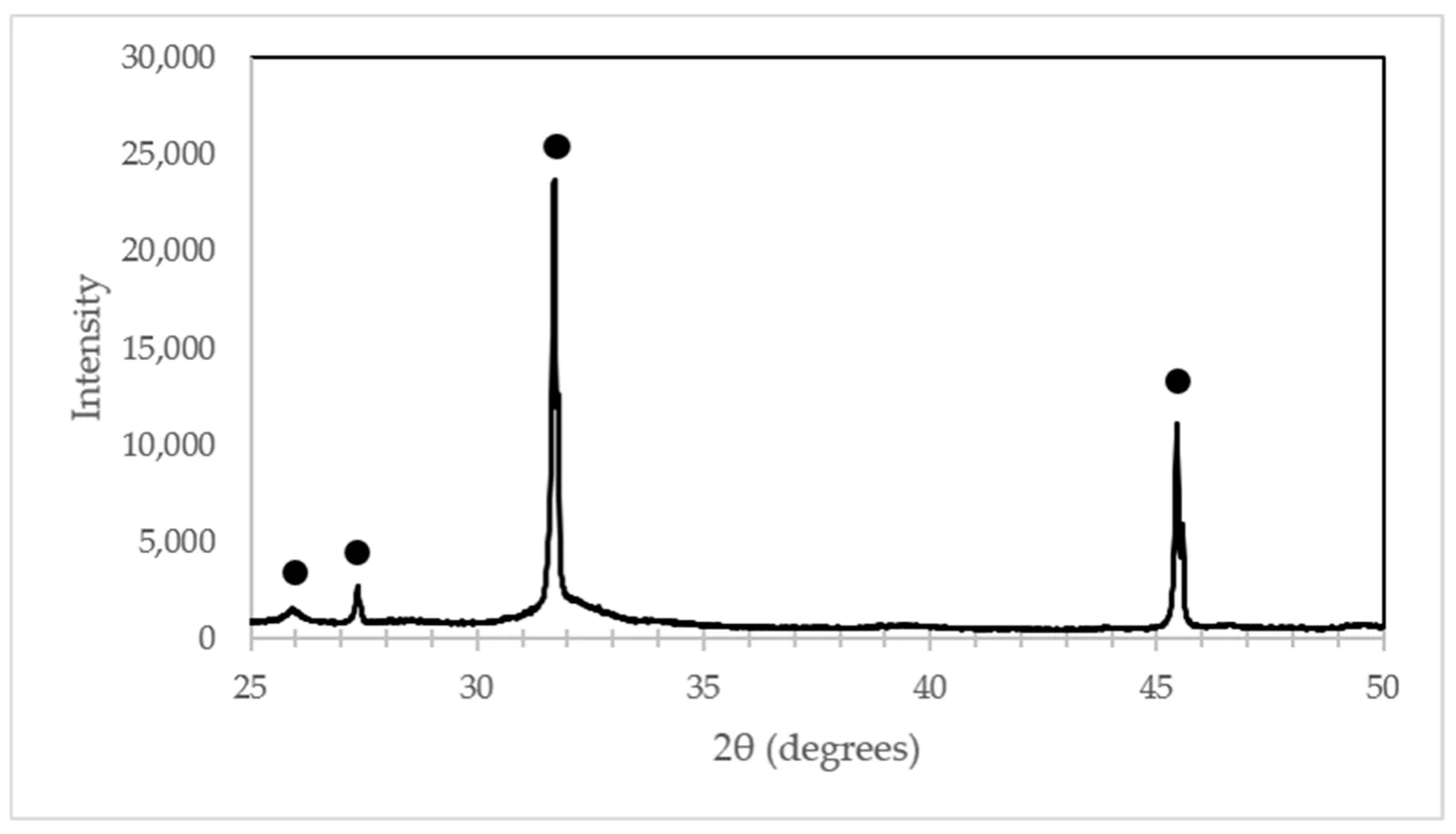
XRD of calcium phosphate shell particles, with black dots marking major peaks for crystalline and amorphous calcium phosphate composition at ~25.9, ~27.4, ~31.7 and ~45.4 degrees.

XRD analysis of the synthesized CaP microshells revealed characteristic peaks at approximately 25.9°, 27.4°, 31.7°, and 45.4° 2θ, corresponding to the (002), (102), (211), and (203) planes, respectively, which may be ascribed to hydroxyapatite (Figure 2). The broad diffuse background region between 30° and 33° 2θ is indicative of the presence of amorphous mineral phases.

### 3.2. Determination of Silane Deposition on Titanium Samples

**Figure 3:**
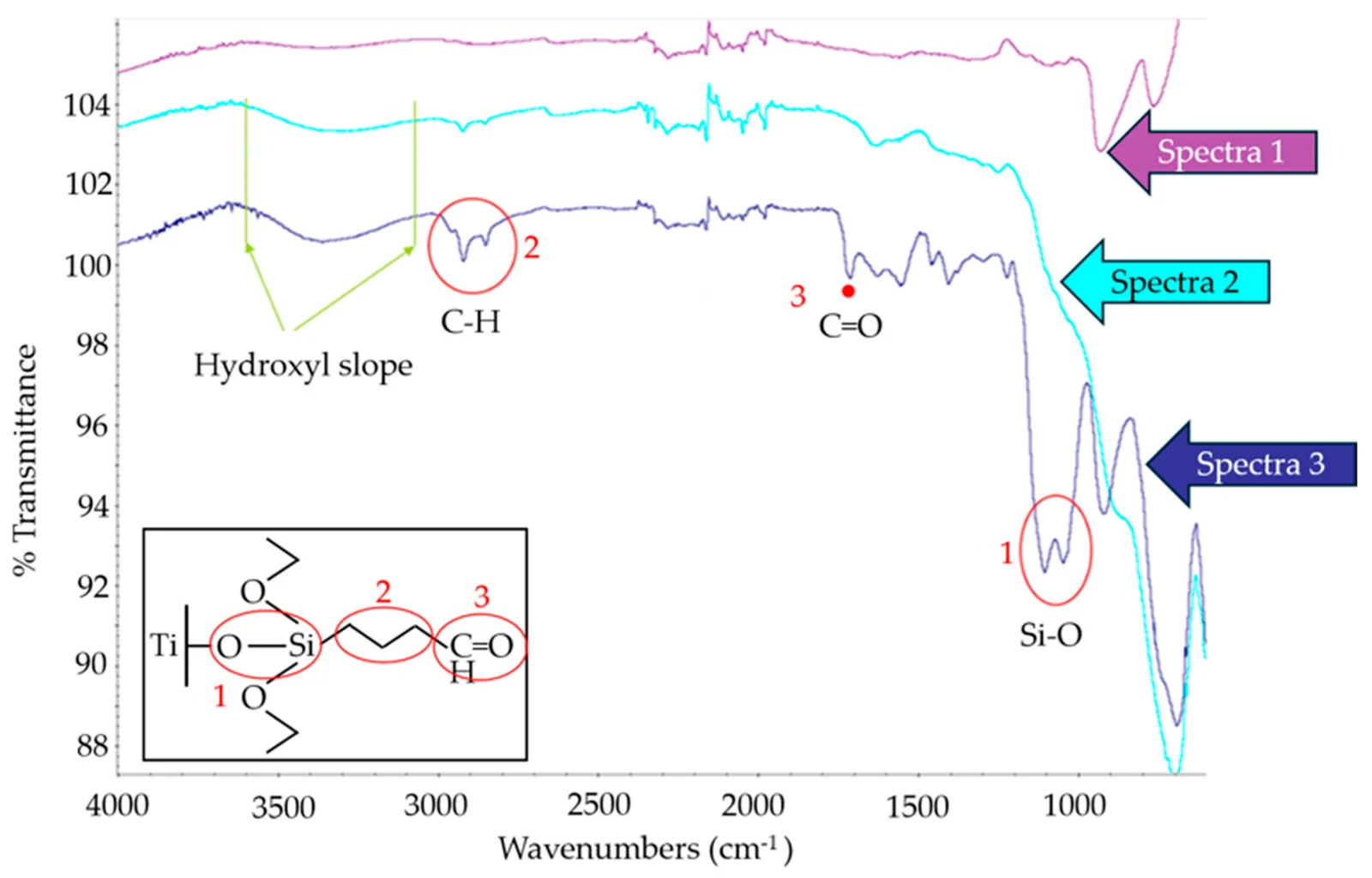
FTIR analyses of surface chemistry and deposition of silane on Ti in preparation for the electrospray chitosan coating process. Spectra 1 corresponds to cleaned Ti. Spectra 2 shows a noticeable strong t broad peak at 3100 and 3600 cm^−1^, indicating enhancement of -OH groups on surface after NaOH treatment. Spectra 3 corresponds to the silanized Ti surface with the peaks at ~1100 cm^−1^ (circle labeled 1), at ~2850 cm^−1^ (circle labeled 2) and at ~1720 cm^−1^ (dot labeled 3) indicating the silane Si-O bond, and -CH_2_, and C=O (aldehyde) groups respectively in the chemical structure (inset).

The FTIR analysis of silane deposition on the Ti surfaces is shown in Figure 3. In the figure, spectra 1 is the FTIR of the cleaned Ti surface. In spectra 2, after NaOH treatment of the Ti surface, there is a noticeable increase in the broad peak between 3100 and 3600 cm^−1^ and in the peak at 1555–1640 cm^−1^, corresponding to stretching and bending of -OH groups, indicating an increase in the presence of -OH groups on the Ti surface. In spectra 3, the absorbances of -CH_2_, C=O (aldehyde), and Si-O- were detected by peaks at 2925–2850, 1720, and 1200–1000 cm^−1^, respectively, indicating successful deposition of the silane onto to the Ti surface.

### 3.3. Refinement of Electrospraying Parameters

**Figure 4:**
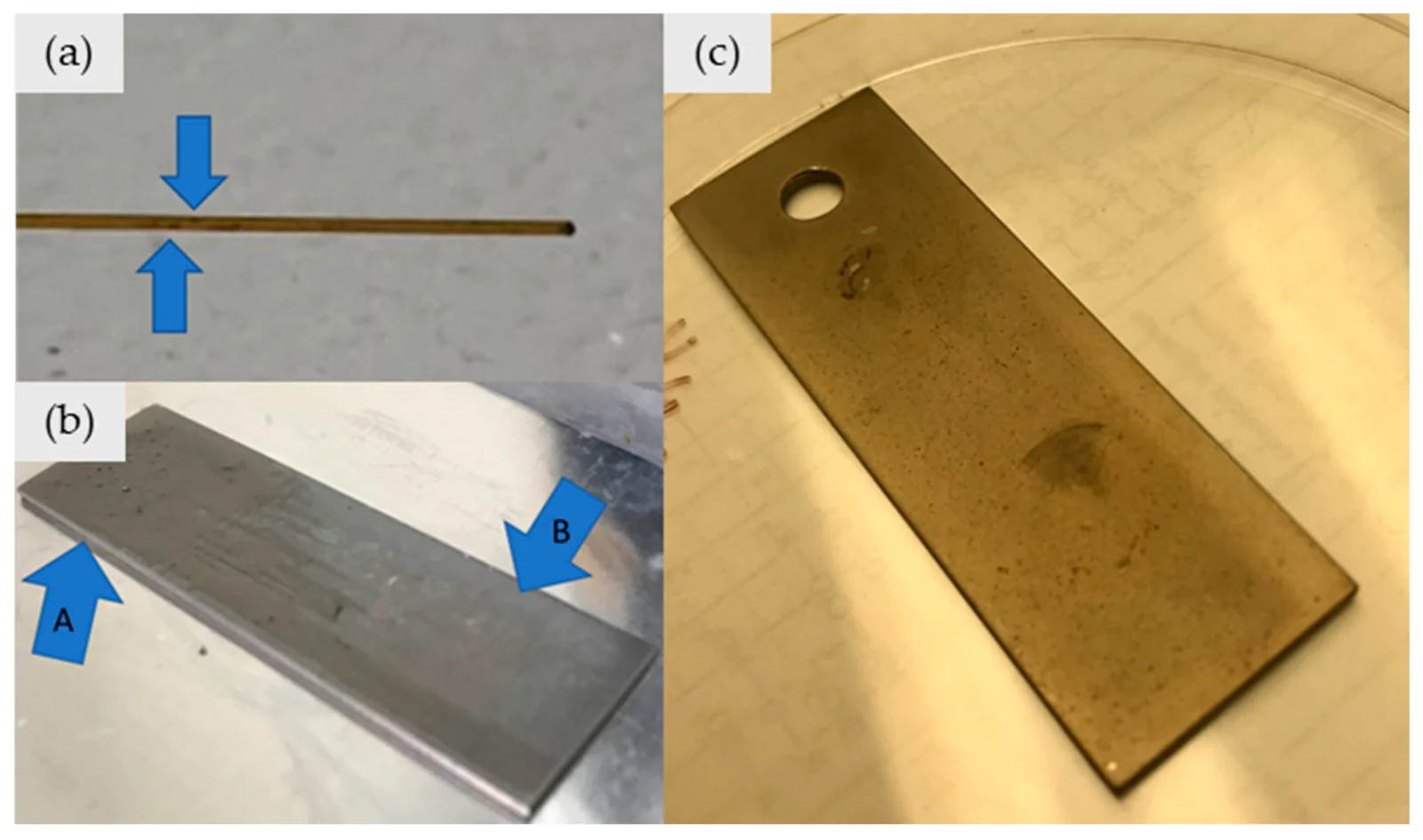
(**a**) A 250 µm capillary tube with arrows indicating CaP aggregate clogs. (**b**) Titanium sample for which the coating process stopped due to capillary clogging mid-spray. Arrow A indicates coating area, arrow B indicates uncoated area. (**c**) Successful chitosan–CaP coating electrosprayed at 6.3 kV using a 320 µm capillary tube, approximately 13 psi and a substrate–capillary distance of 3 mm.

Initial electrospraying using a 250 µm capillary (Table 1) produced clogging when solutions contained ≥0.25 wt% CaP, which resulted in incomplete coatings (Figure 4b). Aggregation was attributed to CaP particle accumulation along the capillary walls. Increasing the capillary diameter to 320 µm eliminated visible clogging and enabled consistent coating formation up to 1.0 wt% CaP.

Because the larger capillary increased solution throughput, pressure was reduced from 45 psi to 15 psi to recover a stable spray cone and then to 13 psi to prevent substrate flooding during longer spray durations.

Substrate–capillary distance (2–7 mm) and applied voltage (4–8 kV) were also evaluated, but variations within these ranges did not produce discernible improvements in coating quality. The final optimized parameters yielded uniform coatings (Figure 4c) and are summarized in Table 2.

### 3.4. Determination of Surface Morphology and Composition of Chitosan–CaP Coatings

**Figure 5:**
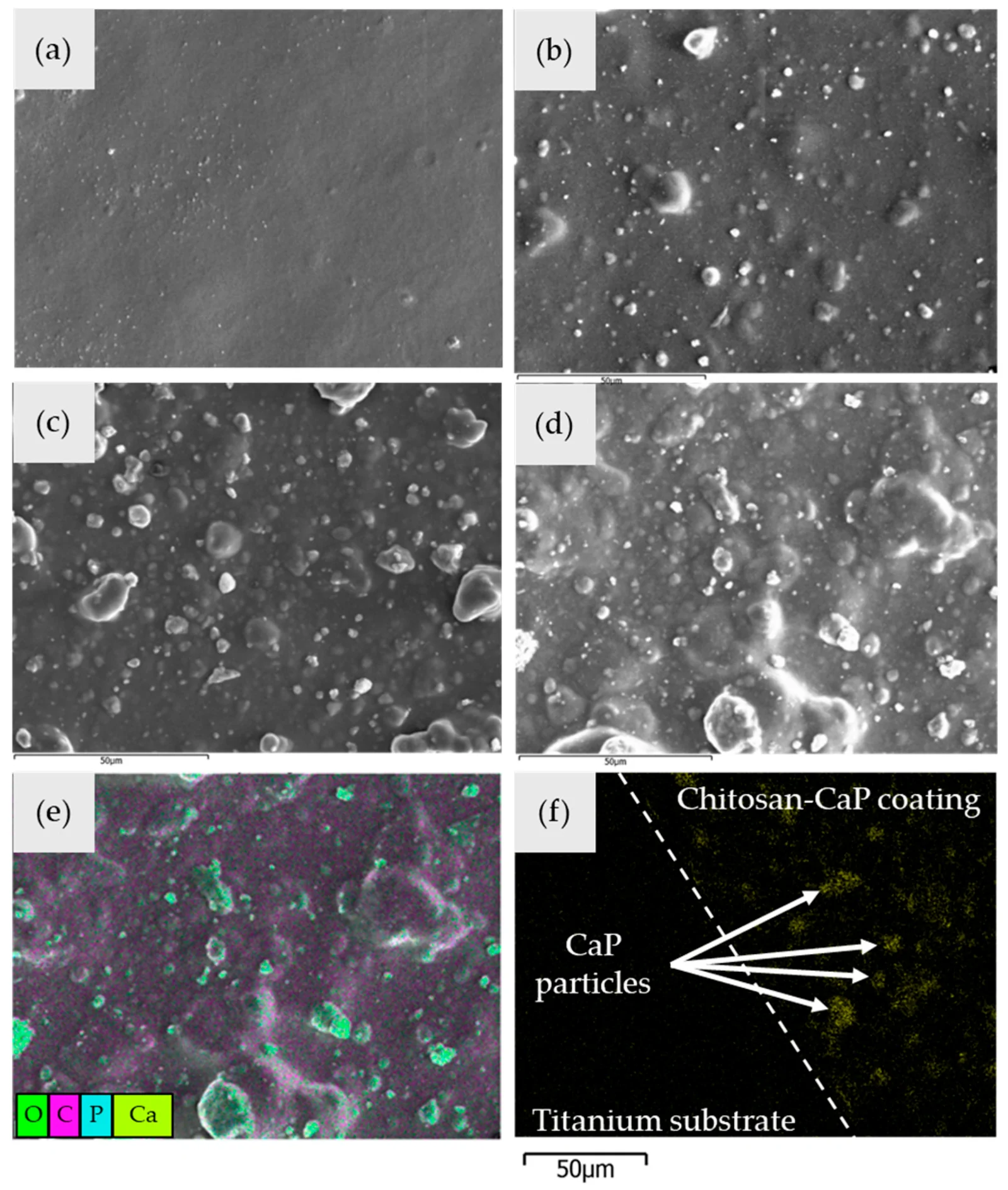
Representative SEM images showing surface morphology of electrosprayed chitosan coatings with (**a**) 0 wt% CaP shells, (**b**) 0.25 wt% CaP shells, (**c**) 0.5 wt% CaP shells, and (**d**) 1.0 wt% CaP. (**e**) False-color EDS map overlay on SEM image of chitosan coating with 1.0 wt% CaP shells showing distribution of CaP shells (green) in chitosan (pink) coating. (**f**) Cross-section EDS mapping chitosan coating with 1.0 wt% CaP shells showing Ca particles (green) distributed throughout coating thickness (dotted line indicates interface between substrate and coating).

All chitosan–CaP coatings showed more rough textured morphology in the SEM images than the control chitosan coating (Figure 5a–d). Qualitatively, as CaP shells increased from 0.25 to 1 wt%, the coating texture increased, reflecting the increase in CaP particle content. EDS mapping, with false-color overlay of Ca and P, showed a visually uniform distribution of particles in the coating surfaces (Figure 5e). EDS mapping for Ca of the cross-section of the coatings also revealed CaP particles throughout the thickness of the coatings (Figure 5f).

### 3.5. Determination of Bond Strength of the Chitosan Coating

Results of tensile testing (Table 3) demonstrated that all electrosprayed coatings on silanized Ti surfaces exhibited statistically significantly greater mean tensile bond strengths than coatings made on non-silanized Ti surfaces (*p* < 0.05). There was no statistically significant difference between the 0.25 wt% CaP, 0.5 wt% CaP, 1.0 wt% CaP, and control (0 wt% CaP) chitosan coatings electrosprayed onto silanized Ti. Similarly, there was substantial overlap in the 95% confidence intervals of bond strengths for all electrosprayed coatings on silanized surfaces, indicating no statistical difference between coatings. However, there was no overlap in the 95% confidence interval of coatings on non-silanized surfaces with coatings on silanized surfaces, indicating that all coatings on silanized surfaces had significantly greater adhesion than coatings on non-silanized Ti surfaces.

**Figure 6:**
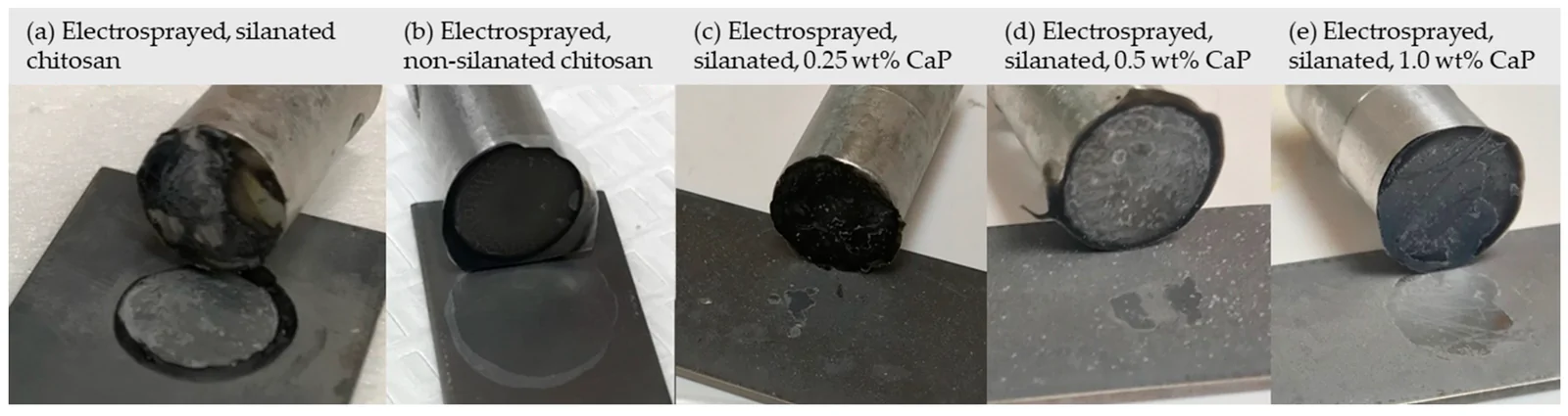
Coatings after tensile test failure. (**a**) Electrosprayed, silanized, no loaded CaP; (**b**) electrosprayed, non-silanized, no loaded CaP; (**c**) electrosprayed, silanized, 0.25 wt% loaded CaP; (**d**) electrosprayed, silanized, 0.5 wt% loaded CaP; and (**e**) electrosprayed, silanized, 1.0 wt% loaded CaP.

Visual examination of the pins and test coatings after tensile testing revealed that the control (0 wt% CaP) electrosprayed coatings on silanized Ti surfaces remained predominantly adhered to the substrate, with some cohesive failure of the chitosan coating observed (Figure 6a). The electrosprayed coatings on non-silanized Ti surfaces showed complete loss of coating adhesion at the coating–Ti substrate interface (Figure 6b). A progressively greater failure of adhesion to the substrate was observed as the wt% of CaP particles increased from 0.25 wt% to 1.0 wt% in the chitosan coatings (Figure 6c–e).

### 3.6. Determination of Hydrophilic Nature of Chitosan Coating

The results of the water contact angle measurement on electrosprayed chitosan coatings are shown in Table 4. All test chitosan coatings exhibited water contact angles less than 90°. Statistical analyses indicated that there was no significant difference in water contact angles between any of the coatings.

### 3.7. In Vitro Evaluation of Chitosan Coatings

**Figure 7:**
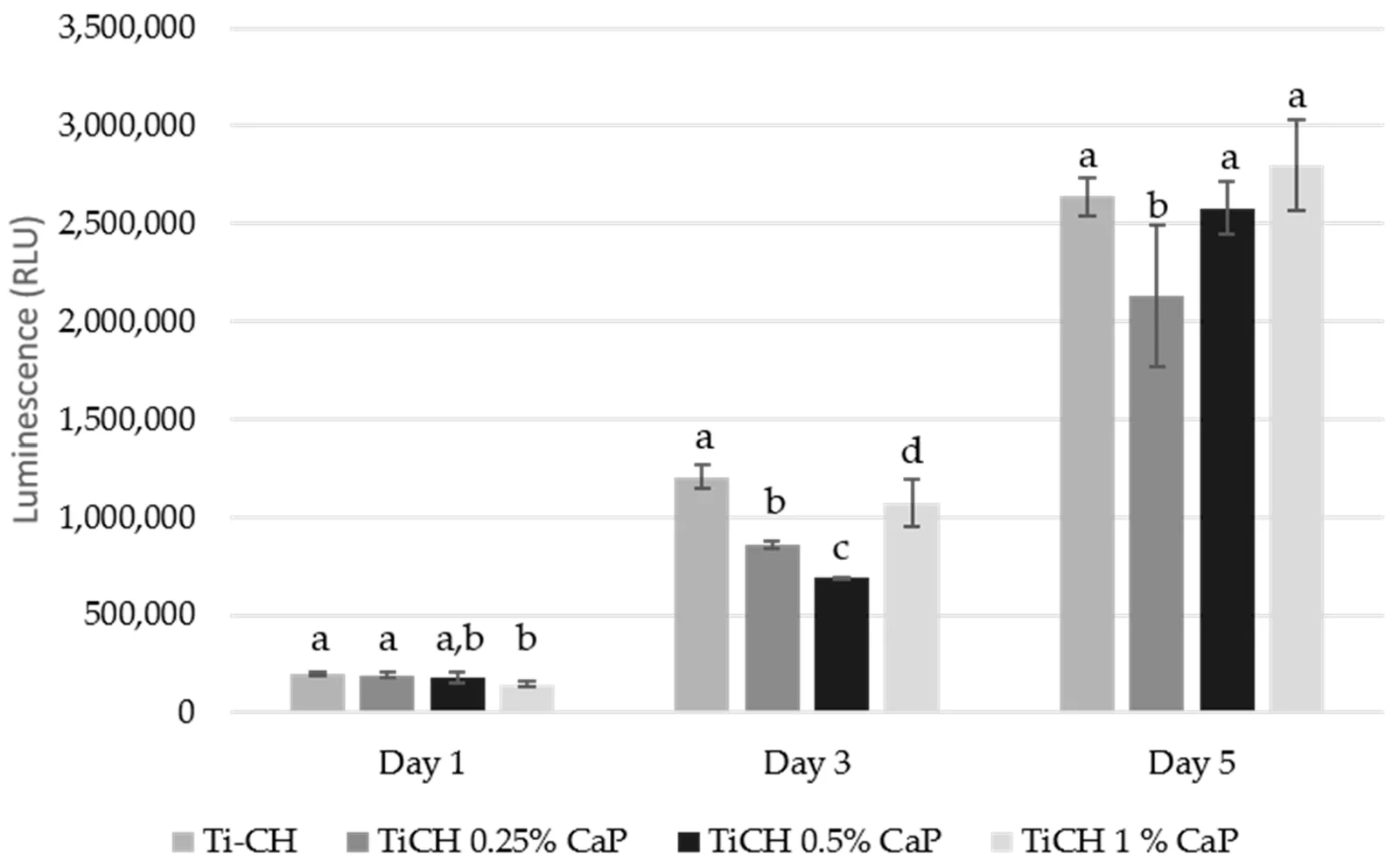
In vitro growth study on chitosan-electrosprayed Ti implant samples using W-20-17 BMSCs. Bars represent average ± standard deviation (*n* = 4). The same letters indicate values that are statistically similar to other coatings on the same day.

The results for the in vitro viability and growth of cells on the test chitosan coatings and the control Ti are shown in Figure 7. All groups showed increases in cell number over time based on increases in luminescence. Statistical analyses using two-factor ANOVA indicated statistically significant differences in cell growth among the different test coatings (*p* = 4 × 10^−30^), significant differences over time (*p* = 8 × 10^−9^), and a significant interaction between factors of test coatings and time (*p* = 6 × 10^−7^). Due to interactions, data were analyzed separately based on days.

Statistical analyses at day 1 indicated that initial cell growth based on luminescent measurements was similar for the control (0 wt% CaP), 0.25 wt% CaP and 0.5 wt% coatings, and both the control and 0.25 wt% CaP coatings were significantly greater than either the tissue culture plastic control or the 1.0 wt% CaP coatings. On day 3, all coatings were statistically different from each other, with TCP having the greatest cell growth, followed by the control (0 wt% CaP) coatings, the 1.0 wt% CaP chitosan coatings, and then the 0.25 wt% and 0.5 wt% CaP–chitosan coatings. At day 5, the TCP control still exhibited the statistically greatest cell growth as compared to all the test chitosan coatings. The 0.25 wt% CaP chitosan coatings showed the statistically lowest cell growth at day 5 compared to TCP and other test chitosan coatings, but there were no statistical differences detected between the control (0 wt%), 0.5 wt%, and the 1.0 wt% CaP chitosan coatings. All groups showed a net increase in viable W-20-17 cells over the 5-day culture period.

## 4. Discussion

Chitosan and calcium phosphate have both been used to manufacture Ti implant coatings in part to their biocompatibility, osteoconductivity, and potential to improve early bone–implant interactions [14,15,16,20,21,22,23,24,25,26,27,28,29]. However, many coating technologies are difficult to use on complex 3D surfaces, resulting in material waste, or they can damage incorporated bioactive components. Electrospraying addresses several of these limitations by enabling deposition onto complex surfaces, processing under relatively mild conditions, and reducing material waste [26,33,34,35]. The present study was designed as an initial materials and coating characterization investigation to establish an approach prior to pursuing further studies including in vivo osseointegration evaluations. Its primary novelty is the integration of CaP microshell structures into an electrosprayed chitosan coating bonded to silanized Ti with an evaluation of coating morphology, particle distribution, wettability, adhesion strength, and preliminary bone cell cytocompatibility.

The microshells synthesis produced particles with sizes and a morphology comparable to those reported by Wu et al. [38]. Carbon microspheres acted as sacrificial templates for CaP deposition and were removed during thermal treatment, generating shell-like particles. EDS confirmed the Ca and P composition of the particles, and XRD indicated that the particles contained both a crystalline phase and amorphous phase. These findings, together with the rough, lath-like apatite morphology reported by Wu et al. [38], support the formation of a partially crystalline apatite-like phase that is typical of biologically derived bone-like mineral [40]. Because natural bone mineral is only partially crystalline, this mixed-phase composition more closely reflects the mineral structure of native bone than highly crystalline hydroxyapatite. The coexistence of amorphous calcium phosphate and crystalline hydroxyapatite-like mineral may therefore provide a more biomimetic surface, potentially enhancing osteoconductive interactions and supporting osseointegration.

The particles exhibited a zeta potential within a range associated with aggregation and flocculation due to van der Waals attractive forces [41], which, along with particle size, contributed to the observed clogging and poor coating deposition during the electrospray process. This issue was corrected by increasing the capillary tube diameter to 350 µm and reducing the electrospraying pressure to 11–15 psi. These adjustments enabled a stable and reproducible electrospray process that produced uniform coatings without capillary clogging or substrate flooding. SEM/EDS analyses of coating surfaces and cross-sections revealed homogenous distribution of CaP particles throughout the electrosprayed chitosan coatings. This uniform distribution provides a more continuous osteoconductive mineral phase across the coating surface and minimizes localized particle agglomeration, thereby supporting consistent cell–material interactions that may promote osseointegration. SEM observations indicated that the surface roughness of the coatings increased with the wt% of CaP particles. The increase in roughness was also reflected in the increase in variability in water contact measurements of the coatings with CaP particles as compared to the plain chitosan coatings [42]. An increase in coating texture may be beneficial because rough surfaces are generally associated with increased BMSC attachment, growth and osseointegration [43,44]. Several studies have indicated that moderately rough surfaces with surface roughness values (Sa) of between 1.0–2.0 microns result in better osseointegration of dental implants than implants with lower or higher surface roughness. A limitation of this study is that the surface roughness was not measured quantitatively [45,46]. Nevertheless, these results show that it may be possible to adjust CaP particle concentrations to target moderately rough surface values for the electrosprayed chitosan–CaP coatings to enhance osseointegration.

Overall, these results demonstrated that by adjusting parameters, uniform CaP–particle–chitosan composite coatings were able to be made using the electrospraying process. Additionally, the apparent roughness of the coatings can be modified by changing the wt% of CaP particles in the electrospray solution.

In this work, the silanol group generated from hydrolyzed TESBA molecules reacted with -OH groups on the Ti surface to form covalent Si-O-Ti linkages, while the aldehyde end group could react with amino groups in the chitosan coating [22,26]. Previous studies reported that bonding of chitosan coatings is improved by treating the Ti with a strong base to create an -OH-rich surface that provides more sites for bonding of the silane linker molecule [22]. The FTIR analyses confirmed the development of an -OH-rich surface on the Ti after alkali treatment and subsequent attachment of silane molecules and formation of Si-O bonds on the Ti surface. Bonding of the coatings was demonstrated by an approximately two-fold increase in the adhesive bond strength of electrosprayed coatings on silane-treated Ti surfaces compared with non-silanized coatings. The complete loss of coating adhesion at the Ti–coating interface reflected the low coating adhesion strength to Ti surfaces without the silane linker molecule. In contrast, mixed cohesive/adhesive failure was observed at the Ti–coating interface for electrosprayed coatings on silanized Ti surfaces, indicating that silane-mediated bonding occurred and increased coating adhesion strengths to the Ti surface. These results are similar other studies and demonstrate the potential for using silane molecules to improve adhesive bond strengths of chitosan coating [20,21,22,26].

The electrosprayed coatings on the silanized Ti surface exhibited adhesive bond strengths between 4.31 to 5.62 MPa, and no statistically significance differences were observed among the silanized coatings with 0–1.0 wt% CaP. These bond strengths are similar those reported by Chng et al. for electrosprayed coatings as with prior solution-cast chitosan coatings [20,26]. More broadly, coating adhesion and delamination resistance are central performance considerations for multifunctional orthopedic bioactive coatings [47]. Thus, the 4–6 MPa range observed here suggests that incorporating CaP microshells up to 1.0 wt% did not statistically compromise adhesion within the tested formulation range. Although the mean bond strength decreased numerically with increasing CaP loading, the differences were not statistically significant and should not be interpreted as approaching significance. The visual failure pattern showed a greater adhesive failure component as the CaP content increased, which may indicate that CaP particles at or near the Ti–coating interface partially interrupt silane-mediated bonding to chitosan. Further increases in CaP loading may therefore reduce coating adhesion and should be evaluated before use in mechanically demanding orthopedic applications.

All coatings had water contact angles less than 90°, indicating that the coatings were hydrophilic. This agrees with prior studies of chitosan-coated surfaces [22,23,24,26,48]. Contact angle measurements did not identify any differences in hydrophilic properties between the plain chitosan coatings and the coatings containing CaP particles. It is possible that the chitosan could be effectively covering the surface of the coatings, hindering major effects that the CaP shells would have on the coatings’ wettability. However, it was also observed that addition of CaP microshells in the chitosan coating exhibited an increase in variability in water contact angle measurements. Surface roughness can lead to an increase in scattering of water contact angle measurements as the drops are likely to be pinned during deposition [42]. The increase in surface roughness due to the presence of the CaP microshells may therefore have contributed to an increase in variability in water contact angles, reducing ability to identify any statistical differences, if any, between the coatings. Regardless, all coatings exhibited strongly hydrophilic characteristics that were favorable to the high degree of cell attachment, as reflected in the day 1 cell culture data, in which higher number of viable cells were generally observed on chitosan coatings as compared to tissue culture plastic controls.

The evaluation of cytocompatibility and cell growth on the electrosprayed coatings indicated that all coating groups supported high cell viability and attachment at day 1 and robust cell growth over the 5-day culture period. High BMSC viability and attachment are generally considered to be good predictors of osteocompatibility [49,50]. However, statistically significant differences in cell growth were observed between coatings based on CaP wt% at days 3 and 5. No clear trend in cell growth was identified across CaP concentrations, although both the plain chitosan (0 wt% CaP) and 1.0 wt% CaP coatings supported greater cell growth than the 0.25 wt% and 0.5 wt% CaP groups at later time points. It is unclear why the 0.25 wt% and 0.5 wt% CaP coatings exhibited reduced growth at later time points, particularly given their comparable or higher viability on day 1. A limitation of the present study is that calcium ion release and quantitative surface roughness were not measured, both of which may influence cell proliferation. Calcium concentration is known to modulate BMSC proliferation and differentiation, with higher concentrations generally promoting cell growth [50]. Additionally, surface roughness can influence bone cell behavior [43,44,49,50]. Further studies are needed to elucidate the interplay between calcium ion release, surface roughness, and surface chemistry in regulating BMSC growth and differentiation on these coatings.

## 5. Conclusions

This research demonstrated that electrospraying techniques were successfully used to create a chitosan–calcium phosphate coating bonded via silane molecules to titanium implant material surfaces. The updated electrospraying technique incorporated up to 1.0 wt% of CaP into chitosan solutions while maintaining sprayability and forming visually uniform coatings. Silanization improved coating adhesion relative to non-silanized Ti, and CaP incorporation up to 1.0 wt% did not produce statistically significant loss of adhesion strength. The hydrophilic nature of the coatings contributed to the high initial cell attachment and viability. While there were some differences in the growth of BMSCs on the coatings with different CaP microshell concentrations, it was judged that all coatings supported BMSC growth over the five days of culture. Overall, these findings support the feasibility of electrosprayed chitosan–CaP microshell coatings as cytocompatible, adhesive bioactive coating platforms for Ti implant materials within the tested proof-of-concept scope.

## Figures and Tables

**Table 1:** Original electrospraying parameters for chitosan.

Parameter	Value
Capillary size	250 µm
Capillary material	Silica
Voltage	6.3 kV
Pressure	45 psi
Substrate–capillary distance	3 mm
Base chitosan solution	3:1 (95% DDA, 1% wt chitosan 0.5% acetic acid + ethanol)

**Table 2:** Revised electrospraying parameters for chitosan loaded with CaP microshells.

Parameter	Value
Capillary size	320 µm
Capillary material	Silica
Voltage	6.3 kV
Pressure	11–15 psi
Substrate–capillary distance	3 mm
Base chitosan solution	3:1 (95% DDA, 1% wt chitosan 0.5% acetic acid + ethanol)

**Table 3:** Tensile bond strength (MPa) of electrosprayed coatings with and without calcium phosphate microshells. Mean bond strength values with different letter superscripts are significantly different (*p* < 0.05). The same letters correspond to values that are statistically similar.

Specimen	Mean Bond Strength (MPa) ± Std Dev	95% CI Bond Strength (MPa)
Electosprayed Non-Silanized (*n* = 16)	2.51 ± 0.96 ^a^	2.04 to 2.98
Electosprayed Silanized (*n* = 12)	5.62 ± 1.70 ^b^	4.66 to 6.59
Electosprayed Silanized + 0.25 wt% CaP (*n* = 6)	5.49 ± 1.34 ^b^	4.42 to 6.56
Electosprayed Silanized + 0.5 wt% CaP (*n* = 6)	4.97 ± 0.92 ^b^	4.23 to 5.71
Electosprayed Silanized + 1.0 wt% CaP (*n* = 5)	4.31 ± 0.68 ^b^	3.71 to 4.91

**Table 4:** Results of the water contact angle measurements (*n* = 4/group) on the test electrosprayed chitosan coatings.

Water Contact Angle Measurements		
Samples (*n* = 4/Group)	Average ± Standard Deviation [% CV]	95% CI
Ti + CH	71.6 ± 1.3° [1.8%]	70.3 to 72.9°
Ti + CH + 0.25 wt% CaP	68.0 ± 4.8° [7.1%]	63.2 to 72.7°
Ti + CH + 0.5 wt% CaP	74.7 ± 3.6° [4.8%]	71.2 to 78.2°
Ti + CH + 1.0 wt% CaP	69.6 ± 4.6° [6.6%]	65.1 to 74.1°

## Data Availability

The original contributions presented in this study are included in the article. Further inquiries can be directed to the corresponding authors.

## Abbreviations

The following abbreviations are used in this manuscript:

ATR	Attenuated Total Reflectance
BMSCs	Bone Marrow Stromal Cells
CAM	Contact Angle Measurement
CaP	Calcium Phosphate
CDC	Center of Disease Control
DI	Deionized Distilled Water
DMEM	Dulbecco’s Modified Eagle’s Medium
EDS	Energy Dispersive X-ray Spectroscopy
FBS	Heat-Inactivated Fetal Bovine Serum
FTIR	Fourier Transform Infrared Spectroscopy
MPa	Mega Pascals
NaOH	Sodium Hydroxide
SEM	Scanning Electron Microscope
TESBA	Tri-Ethoxy-SilylButyrAldhyde Silane
Ti	Titanium
*v*/*v*	Volume per Volume
